# First Italian experience using the automated craniofacial gestalt analysis on a cohort of pediatric patients with multiple anomaly syndromes

**DOI:** 10.1186/s13052-022-01283-w

**Published:** 2022-06-13

**Authors:** Giulia Pascolini, Mauro Calvani, Paola Grammatico

**Affiliations:** 1 Medical Genetics, Department of Molecular Medicine, Sapienza University, San Camillo-Forlanini Hospital, Circonvallazione Gianicolense 87, 00152 Rome, Italy; 2 Pediatrics Division, Woman-Child Department, San Camillo-Forlanini Hospital, Rome, Italy

**Keywords:** Craniofacial, Syndromes, DeepGestalt, Malformation

## Abstract

**Background:**

In this study, we used the novel DeepGestalt technology powered by Face2Gene (FDNA Inc., MA, USA) in suggesting a correct diagnosis based on the facial gestalt of well-known multiple anomaly syndromes. Only molecularly characterized pediatric patients were considered in the present research.

**Subjects and methods:**

A total of 19 two-dimensional (2D) images of patients affected by several molecularly confirmed craniofacial syndromes (14 monogenic disorders and 5 chromosome diseases) and evaluated at the main involved Institution were analyzed using the Face2Gene CLINIC application (vs.19.1.3). Patients were cataloged into two main analysis groups (A, B) according to the number of clinical evaluations. Specifically, group A contained the patients evaluated more than one time, while in group B were comprised the subjects with a single clinical assesment. The algorithm’s reliability was measured based on its capacity to identify the correct diagnosis as top-1 match, within the top-10 match and top-30 matches, only based on the uploaded image and not any other clinical finding or HPO terms. Failure was represented by the top-0 match.

**Results:**

The correct diagnosis was suggested respectively in 100% (8/8) and 81% (9/11) of cases of group A and B, globally failing in 16% (3/19).

**Conclusion:**

The tested tool resulted to be useful in identifying the facial gestalt of a heterogeneous group of syndromic disorders.

This study illustrates the first Italian experience with the next generation phenotyping technology, following previous works and providing additional observations.

## Introduction

Multiple anomaly syndromes can be frequently diagnosed in a clinical setting. They can be characterized by variable congenital defects (heart, genitourinary, central nervous system, and skeletal malformations) and craniofacial anomalies, which can appear distinctive, directing clinical diagnosis, or sometimes subtle or non-specific. Clinical flow-charts can be used to drive the diagnostic process in syndromic patients [1]. However, physicians could be supported in the diagnostic process by the recent computer-aided facial analysis present in Face2Gene (FDNA Inc., Boston, MA, USA; https://www.face2gene.com), powered by the DeepGestalt algorithm. This tool analyzes two-dimensional (2D) facial patient’s images and automatically elaborates a prioritized list of syndromes with similar morphology. Also, the tool computes clinical findings annotated in the form of HPO (Human Phenotype Ontology) terms and adds these to results obtained by the DeepGestalt algorithm.

We experimented with this technology on a selected cohort of pediatric patients with different syndromes and a molecular diagnosis, representing the first study from a single Italian clinical center.

## Subjects and methods

Clinical portraits of 19 pediatric patients with a multiple anomaly syndrome evaluated at the main involved Institution (Medical Genetics, San Camillo-Forlanini Hospital of Rome, SCFH) by the first author (GP), were undertaken in Face2Gene from 2019 to 2020. The study enrolled only patients i) with a molecularly confirmed diagnosis ii) for whom good facial photographic documentation was available iii) affected by a syndrome with a validated facial model available in the platform. The selection method was mainly influenced by the fact that not all syndromic patients had a molecular diagnosis or by the denial of consent to obtain photographic material or by the poor quality of images, which resulted to be not suitable for the facial analysis by the tool, in addition to the presence of a validated facial model in the tested system. Moreover, it must be considered that not only pediatric patients affer to a clinical genetics service, explaining the not so wide number of the subjects included in the present research.

The study was focused on the pediatric age cause an early correct diagnosis can reveal important for the clinical and therapeutical management of the patient.

**Fig. 1:**
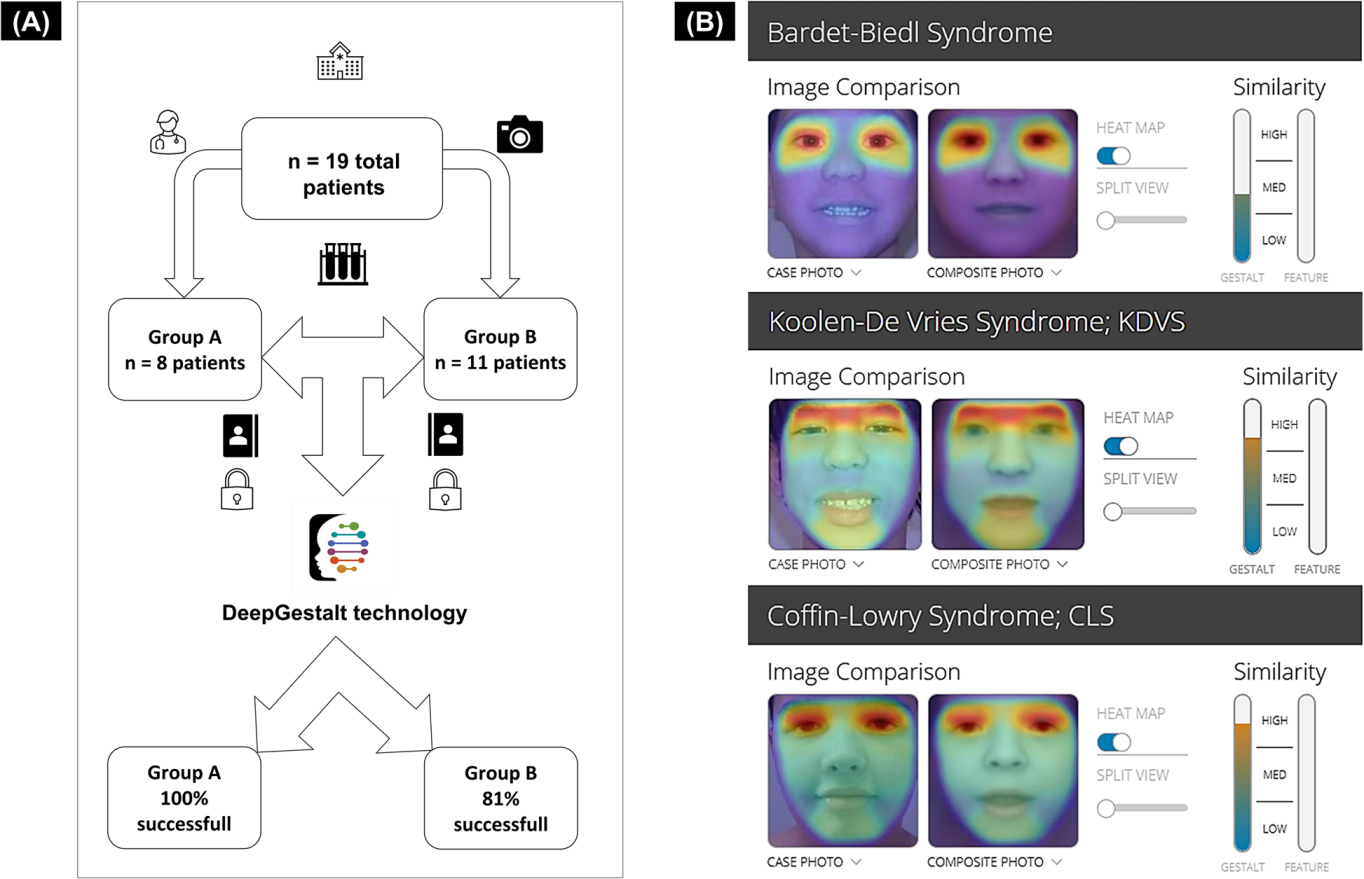
**A** A diagram demonstrating the main structure of the present study **B** Facial analysis results using the heat-map function of the DeepGestalt technology (CLINIC application) for some of considered conditions: from top to bottom Bardet-Biedl 1, Koolen de-Vries, and Coffin-Lowry syndromes. Hot colours represent the most facial region overlap

The study cohort comprised pediatric individuals (aged 3 months-14 years) of Caucasian ethnicity. Studied patients were affected by monogenic diseases (14 individuals) and chromosome aberrations (5 patients), including structural anomalies. Two subjects were siblings. All diagnoses are listed in Table 1. Patients have been categorized into two groups, based on the number of evaluations, since dysmorphisms can change over time: Group A (8 individuals), was evaluated twice at different ages group B (11 subjects), was evaluated once. Informed consent for image use was obtained. All 2D photos were anonymously uploaded to the Face2Gene CLINIC application for analysis (vs.19.1.3). Syndromic conditions automatically computed for each patient (30 syndromes) have been classified into four subsets (top-1 match, within top-10, top-30, and top-0 matches). The first subset contains syndromes with the closest match (in both evaluations for group A). These have been proposed by the tool as the most probable syndromes confirming clinical and molecular findings. The second and the third subsets include conditions respectively suggested as within the top-10 or top-30 matches (in both evaluations for group A). Failure to suggest the diagnosed syndrome was represented by the fourth subset (top-0 match). The study methodology is resumed in Fig. 1 A.

**Table 1:** Multiple anomaly syndromes considered in the present study

**Group A**	**Patients**	**Syndrome**	**Genetic testing**	**Automated facial gestalt analysis**			
				top-1 match (age)	within top-10 matches (age)	within top-30 matches (age)	top-0 match (age)
	1	CLS	MA	2 y, 4 y			
	2	KdVS	GA	3 y, 7 y			
	3	CSS1	MA	8 y, 13 y			
	4	Chromosome 9p deletion	CA		9 y		12 y
	5	MCPH1		4 y	3 m		
	6	KBGS	MA	5 y, 7 y			
	7	NS		14 m, 2 y			
	8	WHSUS		5 y	9 y		
**Group B**	**Patients**	**Syndrome**	**Genetic testing**	**Automated facial gestalt analysis**			
				top-1 match (age)	within top-10 matches (age)	within top-30 matches (age)	top-0 match (age)
	1	CLS	MA	11 y			
	2	CSS1*			20 m		
	3	CSS1*	GA	20 m			
	4	TRPS1		14 y			
	5	MNKES	MA	4 y			
	6	HRTFDS					2 y
	7	KLEFS1	GA				10 y
	8	BBS1		4 y			
	9	BWS		2 y			
	10	KABUK1	MA	12 m			
	11	MFDGA		5 y			

Abbreviations: *siblings; *y *Years, *m M*onths, *MA *Molecular analysis, *GA *Genomic analysis, *CA *Chromosome analysis, *BBS1 *Bardet-Biedl syndrome 1, *BWS *Beckwith-Wiedemann syndrome, *CLS *Coffin-Lowry syndrome, *CSS1 *Coffin-Siris syndrome 1, *KABUK1 *Kabuki syndrome 1, *KBGS *KBG syndrome, *KdVS *Koolen-de Vries syndrome, *KLEFS1 *Kleefstra syndrome 1, *HRTFDS *Hartsfield syndrome, *MCPH1 A*utosomal recessive microcephaly 1, *MFDGA *Mandibulofacial dysostosis with microcephaly, *MNKES *Muenke syndrome, *NS *Noonan syndrome, *TRPS1 *Trichorhinophalangeal syndrome type 1, *WHSUS *White-Sutton syndrome

## Results

In group A, the tool suggests the correct diagnosis as the top-1 match in 87.5% (7/8), as the top-10 matches in 37.5% (3/8), within the top-30 matches in 0%, and as the top-0 match in 12.5% (1/8) (Table 1). In group B, an exact diagnosis as the top-1 match and within top-10 matches was achieved respectively in 72% (8/11) and 9% (1/11) of cases; the tool was not able to correctly recognize facial gestalt in 18% (top-0 match, 2/11). No syndromes were identified within the top-30 matches (Table 1).

## Discussion

In this study one small series of patients with various syndromic disorders and assessed in a single Italian clinical setting has been analyzed through the CLINIC application of the Face2Gene suite. This is a recent computer-aided facial analysis tool, which can be used to confirm a clinical suspicion of a syndrome, especially if this is characterized by distinctive dysmorphic facies, or to help in addressing the diagnosis in case of subtle and hard to identify craniofacial anomalies. It automatically builds patient-specific computational classifiers (syndrome gestalts) by converting a patient’s photo into de-identified mathematical facial descriptors. The patient’s facial descriptor is compared to syndrome gestalts for which the algorithm was previously trained, to obtain a prioritized list of overlapping syndromes, based on morphology similarity (gestalt scores). The methodology of the facial phenotypes identification through the DeepGestalt technology has been recently illustrated [2].

Previously published studies have experimented with the efficiency of this tool in other single clinical centers, although with different approaches. Mishima et al. (2019) [3], analyzed facial gestalt in Japanese patients with genetic syndromes at different ages, concluding that the tool is useful in suggesting candidate syndromes in the examined populations. Zarate et al. (2019) [4], performed subsequent research, concluding that combined data from both studies showed a top 10 sensitivity rate of 86.6% (52/60) in the routine clinical setting for conditions with a validated facial model, exclusively based on facial analysis. After, other authors tested this tool in the routine clinical setting, also in individuals of Asiatic descent, showing the tool’s reliability exclusively based on facial analysis [5, 6].

In the present research, Mendelian and chromosome disorders were included. The tested tool was able to recognize the diagnosed syndrome in all cases of group A (100%, 8/8). This group includes a heterogeneous sample of craniofacial syndromes, in terms of biological cause and phenotypic presentation (Table 1). Group A included autosomal recessive microcephaly type 1 (MCPH1, MIM#251200), a rare craniofacial malformation syndrome, which has been evaluated over time. For this case, the tool identified typical facial anomalies, suggesting the correct diagnosis as the top-1 match at 4 years and within top-10 matches in neonatal age, suggesting a more marked craniofacial appearance with age. Conversely, one patient with chromosome 9p deletion syndrome (monosomy 9p, MIM#158170) was correctly diagnosed at 9 years (within top-10 matches) and not in older age (12 years, top-0 match). Regarding group B, a correct diagnosis was globally achieved in 81% of cases. Conditions that did not receive a suggested diagnosis, are represented by Hartsfield syndrome (HRTFDS, MIM#615456), a very rare malformation condition with holoprosencephaly, ectrodactyly, and cleft/lip palate, and Kleefstra syndrome 1 (KLEFS1, MIM#610253). Our HRTFDS patient lacked cleft/lip palate but displayed dysmorphic ears, which are not specific to this only one condition, being associated with other disorders. The girl with KLEFS1 had atypical craniofacial features which could be related to the other genes included in the small microdeletion involving the 9q34.3 critical region. This points out the possibility to find atypical phenotypes, for which identification is crucial the experience of the clinician.

Interestingly, in the present study, we analyzed several chromatin disorders, such as Coffin-Lowry (CLS, MIM#303600) (Fig. 1 B), Coffin-Siris 1 (CSS1, MIM#135900), Koolen de-Vries (KdVS, MIM#610443), (Fig. 1 B), White-Sutton (WHSUS, MIM#616364), Kabuki (KABUK1, MIM#147920), Kleefstra 1 (KLEFS1, MIM#610253) and KBG syndromes (KBGS, MIM#148050), which are characterized by a similar facial gestalt. All these syndromes were recognized as the highest-ranking syndrome even for images taken at different ages. This highlights the distinction by the system of the similar craniofacial contour of these single conditions.

## Conclusions

This research represents the first DeepGestalt study performed on a cohort of pediatric patients affected by multiple anomaly syndromes recruited in a single Italian clinical center. We used this tool on molecularly confirmed individuals, some at different ages (group A).

Interestingly, several chromatin disorders (37%, 7/19/20) evaluated at different ages have been included. The technology proposed for these conditions the correct diagnosis as the top-1 match in 80% (8/10).

The obtained results could suggest the DeepGestalt technology as good support in recognition of facial gestalt of malformation syndromes in a pediatric clinical setting, according to one recent research in the field [7].

## Data Availability

All data regarding this manuscript are available from the corresponding author upon a reasonable request.

## Abbreviations

2D: two dimensional
MCPH1: autosomal recessive microcephaly type 1
KLEFS1: Kleefstra syndrome 1
CLS: Coffin-Lowry syndrome
CSS1: Coffin-Siris syndrome 1
KdVS: Koolen de-Vries syndrome
WHSUS: White-Sutton syndrome
KABUK1: Kabuki syndrome 1
KBGS: KBG syndrome
